# Factors influencing delayed presentation of periorbital carcinoma requiring orbital exenteration

**DOI:** 10.4102/jcmsa.v3i1.201

**Published:** 2025-08-11

**Authors:** Nadine van Wyk, Akiel Asvat, Mokokomadi A. Makgotloe, Mantoa Mokhachane

**Affiliations:** 1 Division of Ophthalmology, Department of Neurosciences, University of the Witwatersrand, Johannesburg, South Africa; 2 Unit for Undergraduate Medical Education, Faculty of Health Sciences, University of the Witwatersrand, Johannesburg, South Africa

**Keywords:** ophthalmology, exenteration, delayed presentation, periorbital carcinoma, tertiary hospital, qualitative

## Abstract

**Background:**

Ocular surface squamous neoplasia is the most widespread indication for orbital exenteration and the presenting feature of human immunodeficiency virus (HIV) in 50% to 86% of patients in endemic areas. Overall survival at 1 year post-exenteration ranged from 69.1% to 97.0%, according to a review article, with the lower rate attributed to delayed referral.

**Methods:**

A qualitative, descriptive phenomenological study was conducted to identify factors responsible for delayed presentation in advanced periorbital carcinoma requiring orbital exenteration. A total of 25 participants were enrolled using convenience sampling. Data were collected by means of a structured questionnaire and open-ended questions. The questionnaire was conducted as a semi-structured interview and recorded with a voice recorder, and the interviews were coded and themes were identified. Data were analysed using Colaizzi’s seven steps for thematic analysis.

**Results:**

Six major themes were identified: a disregard for the seriousness of the condition; financial constraints; delays caused by the clinic; alternative treatment sought; stigma and fear; and relief and hope.

**Conclusion:**

Participants acknowledged that the mass was ignored. However, once healthcare was sought, many felt treatment was delayed as they had to wait for results or because of delayed appointment dates. A lack of financial freedom and a fear of loss of income further delayed participants. Traditional healers were sought as an adjunct to allopathic medicine to appease family. Stigma surrounding the loss of an eye burdened and alienated participants, but many were able to look to the future and forge a new hope and identity.

**Contribution:**

This study contributes to our understanding of the patient-related and systemic factors leading to delay. It highlights key barriers and and promotes earlier diagnosis and intervention.

## Introduction

Ocular surface squamous neoplasm (OSSN) is the most widespread indication for orbital exenteration (OE) done in adults in Africa.^1^ Ocular surface squamous neoplasm is a broad term that ranges from precancerous to cancerous lesions of the conjunctiva. Lesions can start as conjunctival intraepithelial neoplasia (CIN) to a grossly invasive tumour with bony orbital invasion and intracranial extension.^2^ Ocular surface squamous neoplasm can present either in the elderly male population in temperate climates or, as is the case in sub-Saharan Africa, it is associated with a younger female population.^1^ This switch to a younger population is largely attributed to the human immunodeficiency virus (HIV) pandemic.^1^ Ocular surface squamous neoplasm has been found to be a presenting feature of HIV in endemic areas in 50% to 86% of patients.^3^

The reported incidence of OSSN in sub-Saharan Africa is 1.6–3.4 per 100 000 persons per year. This is compared to the United States (US) and Australia, where the reported incidence is only 0.03–1.9 per 100 000 persons per year.^1,4^ Additional risk factors for the development of OSSN are ultraviolet B (UVB) radiation exposure and human papilloma virus (HPV).^4^

The primary treatment modality is either medical or surgical excision of the entire tumour.^3^ Patients who fail to respond to treatment can undergo plaque brachytherapy.^5^ Failing this, patients will require exenteration of the orbital tissues. Orbital exenteration is an invasive and disfiguring surgery for the management of periorbital tumours.^6^ It involves the complete removal of periorbital tissue, extraocular muscles, orbital contents and fat.^7^

Studies have reported that a proportion of patients in Africa with invasive OSSN first present with an orbital mass that requires OE as the primary procedure. These OEs are often for neglected OSSN.^8^ In a recent review article, the overall survival (OS) rate at 1 year post-OE ranged between 69.1% and 97.0%.^6^ Martel quoted in his article that the lowest OS was reported by Aryasit et al. and they identified delayed referral as the main reason for this. Martel mentioned in his article that the converse was the 97.0% OS rate, as reported by Kuo et al., who attributed the high figure to urgent and aggressive surgical treatment.^6^ Metastatic spread appeared to be the main cause of death.^6^

Being cognisant of the high OSSN rate in sub-Saharan Africa and OSSN being the most widespread indication for OE, it is of the utmost importance to identify which factors are affecting delayed presentation of advanced periorbital carcinoma requiring OE in our hospital patient population. In terms of cancer as a whole, it is well known that delayed presentation is associated with poorer survival, and that reducing delays in diagnosis should be a priority.^9^ The literature suggests that reasons for delayed presentation are influenced by the nature of the symptoms and the awareness of the severity thereof, as well as barriers to healthcare, namely, physical, social or psychological.^9^ Cultural beliefs also lead to delays in seeking medical treatment as they affect how an individual responds emotionally, cognitively and socially to a disease.^10^ A study done in rural Nairobi established that the main barriers to eye services included a lack of perceived need for treatment, financial constraints and some did not know where to receive help from.^11^

A literature review of current cancer prevention found that hinderances to healthcare had similar findings as above-stated, including competing health demands.^11^ An important point to consider is that patients often only consult doctors once symptoms become too distressing and impact day-to-day living. In low socio-economic levels, meeting the basic needs of life is more critical and could lead to less awareness of self and of pressing health issues.^9^ Literacy rates are also indirectly related to socio-economic status^9^ and could be a confounding factor. However, a study conducted at four eye centres in Kenya did not find income or low level of education to be a barrier to health-seeking behaviour.^11^

A study from Rwanda looking at delayed presentation of breast cancer patients found that low levels of education and consulting a traditional healer prior to a nurse were determining factors.^11^ In one paper on an Indian population in Delhi, the authors found that patients thought allopathic medicine would not provide palliation or cure but, seeing as there was no cure, they would try allopathic therapy as a last resort.^10^ A diagnosis of cancer can invoke uncertainty and hopelessness. These fatalistic views coupled with inadequate knowledge can lead to a negative attitude to healthcare in general.^10^

Additional factors to be considered are distance in kilometres from the healthcare facility and costs involved in travel. One study found that being female was a predictor of delay. Women often have household and childcare responsibilities to consider before attending to their health. A study done in Tanzania found that women had to get their husband’s permission prior to going to a hospital.^11^ Many patients are employed in the informal sector, and fear of loss of income during this period of health-seeking behaviour can also be a barrier to receiving timeous treatment.^11^

All this considered, it is of vital importance that the treating physician understands the mindset of a patient with cancer and tries to bridge the knowledge-behaviour gap. In so doing, health-seeking behaviour in the community can be improved and patients can be encouraged to present early and with timeous diagnosis and treatment, so as to benefit from better morbidity and long-term cancer survival.

When taking a look at the statistics published by Dr Raman-Abdulla at St John Eye hospital (SJEH) in Soweto, South Africa, the total number of OEs done in 1 year varied from the lowest, being 17 cases per annum, to the most, being 25 cases per annum, as reported in the period from July 2008 to June 2012.^8^ Comparatively, in a review paper of OE, the four largest studies of OE over more than a decade had a mean annual OE rate per centre of 7.5, 7.5, 7.5 and 8.5.^6^ Our hospital appears to have tripled the amount of the annual OE rates compared to other centres, which piques our interest as to why there is such late presentation at this South African hospital.

### Aim

The aim of this study is to identify factors that are responsible for delayed presentation in advanced periorbital carcinoma requiring OE.

### Research question

The research question for this study is as follows: *What was the lived experience of people with delayed presentation of periorbital carcinoma requiring OE*?

### Objective

The objective of this study is to establish which experiences and situations lead to delayed presentation.

## Research methods and design

### Study design

This qualitative study used descriptive phenomenology to discover and describe the shared lived experiences of people who have had an OE for periorbital carcinoma. Phenomenology is the careful study and description of ordinary mindful experiences of daily life. It is to unearth the essence of everyday experiences. It is a portrayal of things that people experience. Phenomenologists view human existence as significant and interesting because we become cognisant of that lived existence. We chose this study design to best understand our study population, that is, people who delayed seeking healthcare. Remaining true to descriptive phenomenology as was developed by Husserl,^12^ the author wrote down their preconceived ideas about the phenomenon prior to data analysis as a means of bracketing. Bracketing is a process whereby any predetermined ideas and beliefs about a specific phenomenon are identified and by so doing, remove any bias that may impede understanding a phenomenon.^12^

### Setting

Persons aged 18 years and older, who attended the SJEH orbital clinic in Soweto, were invited to participate in the study.

### Study population and sampling strategy

A minimum sample size was set at 25 patients, as this was the largest number of patients seen per annum in the previous OE study done at a SJEH.^8^ This proved to be an adequate sample size, as saturation was reached. Saturation, in accordance with qualitative principles, is when no new data appear.^12^ To recruit study participants, the primary author verbally explained to potential participants with a lived experience the purpose of the study. Convenience sampling^12^ was used to recruit participants. This was the most accessible method for recruiting participants who have the same medical condition, as was found in the orbit’s clinic. To be eligible for the study, all adult patients had to be over 18 years old with a confirmed diagnosis of periorbital carcinoma who had already received or still required OE. Patients who required OE for causes other than a carcinoma – for example, orbital mucormycosis – were excluded.

### Data collection

The data were collected between December 2023 and October 2024 by means of a questionnaire which consisted of structured and open-ended questions. Prior to the interview, participants were given an information letter with details of the study, and this enabled the patients to give informed consent before commencing the interviews. Participants who were unable to read or understand English had the information letter read to them or translated into their language of choice. The questions were presented in a standardised fashion to all participants. The open-ended questions permitted the patients to freely describe their subjective experiences and story which culminated in their delayed presentation.^13^ The questionnaire was recorded with a voice recorder, in the participant’s language of choice, and was later transcribed verbatim. Interviewees whose first language was not English or Afrikaans had the interview translated into English. The primary interviewer also took field notes during the interviews. To preserve participant anonymity, each participant was assigned a number during the interview and transcription phase. Participants received no financial reimbursement for participating in the study.

The interviews were focused on whether the periorbital mass was of concern to the patient and the duration of symptoms prior to seeking healthcare, as well as how many healthcare facilities were attended prior to referral to SJEH. This was done to understand why participants delayed themselves in seeking healthcare. In addition, participants were asked which situations they thought influenced their late presentation and whether alternative treatments were sought. Lastly, participants were asked to express their lived experience of having this advanced periorbital mass. The sample questions used in the interview guide are illustrated in Table 1.

**TABLE 1:** Sample questions from the semi-structured interviews.

Number	Question
1	Was the periorbital mass an urgent concern for you?
2	How long were your symptoms present prior to seeking healthcare?
3	How many healthcare facilities did you attend prior to coming to SJEH?
4	Did you receive any alternative treatment, and if so, which treatment?
5	What situation influenced you to present late with this periorbital mass?
6	What has your experience been in terms of having this advanced periorbital mass?

SJEH, St John Eye hospital.

### Data analysis

Data for this descriptive phenomenological study were analysed using Colaizzi’s seven-step method^12^ for the thematic analysis of data. This was led by the primary author. As the first step, the author started by reading the transcripts, as well as rereading them while listening to the audio-recorded data. In this way, the author was able to engage in and become familiar with the data. Thoughts and observations that were perceived as pertinent to the lived experience were noted in the margin of the text of the transcripts. As this was a small dataset, the author thought it would be more helpful to analyse the data manually with pen and paper using printed copies of the transcripts and field notes. This allowed for a more hands-on experience in immersing in the dataset. In the second step, each transcript was read and important statements that stood out were highlighted. Phrases that described specific feelings or perceptions of the healthcare systems, the culture or themselves were identified to try to better understand the phenomenon being studied. As the third step, these phrases were interpreted and given meaning by coding them with keywords or key phrases. Once the codes had been established, the fourth step was to group the codes that had similar meanings together. In the fifth step of the data analysis, the author sought to find the essence of each theme by summarising the data to depict the lived experience of the participants. In our sixth step, we compared the themes to the original dataset and ensured that the two aligned, rereading and adjusting as needed. In the last (seventh) step, validation of the data with participants was not possible as participants were anonymous and follow-up contact was not possible. However, themes were strongly supported across multiple participants to describe the experiences of people who had delayed presentation requiring OE.

### Ethical considerations

Ethics were approved by the University of the Witwatersrand Research Ethics Committee (medical) in November 2023 (Approval number: M2310112). This study was conducted in accordance with the Helsinki Declaration revised in 2013.

## Results

A total of 25 participants who had had or were awaiting OE for orbital carcinoma were interviewed in this study. Table 2 summarises the socio-demographic characteristics of the participants and responses to closed-ended questions about urgency and referral pathways prior to attending a South African hospital.

**TABLE 2:** Participant descriptions (*N* = 25).

Descriptors	*n*	%
**Gender**		
Male	15	60
Female	10	40
**Age (years)**		
18–30	2	8
31–40	3	12
41–50	7	28
51–60	10	40
> 60	3	12
**Population group**		
Black people	24	96
White people	1	4
Mixed ancestry people	0	-
Indian people	0	-
**Marital status**		
Married	7	28
Single	15	60
Divorced	1	4
Widow	2	8
**Highest level of education**		
Illiterate	2	8
Primary school	17	68
Grade 12	5	20
Higher education	1	4
**Annual income†**		
H1	19	76
H2	3	12
H3	0	-
Other	3	12
**Employment**		
Full time	2	8
Part time	0	-
Unemployed	23	92
**Was the periorbital mass urgent for the patient†**		
Yes	19	76
No	6	24
**Symptom duration (months)**		
< 3	6	24
3–6	5	20
6–12	7	28
> 12	7	28
**Facilities attended**		
None	2	8
< 2	13	52
2–5	10	40
> 5	0	-
**Alternative treatment received**		
Yes	5	20
No	20	80

† , The ability to pay for health services is categorised according to patient income: H1, annual income < R70 000; H2, annual income > R70 000 but < R250 000; and H3: annual income > R250 000.

Six major themes were identified to try to understand the lived experiences of people who had delayed presentation for periorbital carcinoma requiring exenteration. These included a disregard for the seriousness of the condition; financial constraints; delays caused by the clinic; alternative treatment sought; stigma and fear; and relief and hope. Direct quotations that best illustrated the major themes were used as supportive evidence.

### Disregard for the seriousness of the condition

Many participants reported that the mass on their eye started out as something small:

‘… like a pimple, something small here in the corner, like something that was itching.’ (Participant 25)

Many did not think this pimple was of significance and just carried on with their lives without further thought of the matter:

‘It started small and then I just ignored it.’ (Participant 18)

A general notion was that this was a minor issue and that it would go away with time. Especially among the male participants, a more nonchalant attitude was held. One participant said in disbelief that this small pimple would:

‘grow to a point where it was closing the whole eye.’ (Participant 22)

While many noticed the tumour growing and worsening of symptoms as a clear indication to seek help, others overlooked the symptoms until the condition worsened:

‘After I realised that thing is growing, that was the time I went to the hospital. To seek help.’ (Participant 3)

‘When I saw this, another one coming on top of the other one, that’s when I started to think let me go see the doctor.’ (Participant 18)

For others, however, pain was the main motivator to get them to seek medical advice:

‘I was worried about it, but I was not caring for it. Until it’s coming pain.’ (Participant 16)

‘We people only go to the clinic when pain is very, very hard.’ (Participant 25)

For many, this condition appeared in their lives as an ordinary event that did not alter the course of their daily activities, until the presence of symptoms such as ‘running stinking water’ as reported by Participant 25 or by the presence of pain. Despite the tumour increasing rapidly in size, many were astounded that this mundane event would be far more sinister:

‘I’ve never thought it’s something dangerous or that it’s cancer.’ (Participant 17)

### Financial constraints

Financial concerns were one of the most prevalent reasons that patients delayed seeking hospital treatment. Unemployment added to this in various ways, the more common one being that the participant was already unemployed at the time of diagnosis and had to rely on family members for assistance. As one participant said:

‘I was not working that time; I did not have that money, so sometimes the family take me. Other times I did not go.’ (Participant 24)

Job security in the informal labour market can be very unforgiving. One participant’s translator explained:

‘He was not working, so if you’re not working on premises, no work no pay.’ (Participant 5)

One participant was employed in another province and was not earning enough to attend his clinic appointments. This participant delayed seeking health advice as it would mean travelling to another province, transport money and loss of income in the period that he was away:

‘I was working that side and I was earning … I was getting monthly salary. So sometimes I did not have salary … to come this side.’ (Participant 6)

One participant, who lives more than 300 km from the referral hospital, had to travel by taxi from his rural town to the district hospital and then only board the government hospital transport bus. The taxi money and the money he would need for food on the trip were too costly for him:

‘I did not have money … you go with the taxi to the hospital sick bay … if I eat on the road when I come … I need to catch a taxi.’ (Participant 1)

Many had to rely on family members, namely, children and younger siblings, to provide transport money to attend the hospital. One participant was the sole survivor of his family, lived alone in Johannesburg and had no means of income. He lamented that food was often a higher priority to spend his money on:

‘You don’t have food; you have nothing; you don’t have work.’ (Participant 9)

This participant also noted that asking neighbours or strangers for money was troublesome as people were reluctant to help you financially:

‘If I need money, then I must borrow from people. I ask them; they say no. Then I say no, you see, if you are alone, if you don’t have family …’ (Participant 9)

Not having money, in addition to the illness, felt like a punishment:

‘You see now, I don’t have money. I’m being punished. I’m suffering. I have nothing …’ (Participant 9)

Job security and being able to provide for their family affected the male participants, especially those who had already undergone surgery and had lost their eye. One participant described that:

‘I’m not working … I was driving and then after this incident, now I can’t see properly.’ (Participant 23)

Unemployment post-exenteration made some participants feel humiliation, as many were breadwinners:

‘I feel ashamed. I’m at home, not working. They [children] ask me for something; I don’t have, because I’m not working.’ (Participant 6)

One participant felt that, had he had the means to get help quickly and efficiently, he would not have had to lose his eye:

‘I was thinking that, maybe if I was having the money to see the specialist, I wouldn’t be having this problem.’ (Participant 24)

Lastly, one patient had already spent an exorbitant amount of money on alternative treatments, and when told they had to see a medical doctor, they were hesitant, as they feared they would have to spend additional money:

‘We didn’t know we must come. Don’t know if you want money … so when they tell us, no go to this doctor, this doctor will give you treatment. Then the money spent on the traditional healer, it was too much …’ (Participant 21)

### Delays caused by the clinic

Given the nature of our healthcare system, with patients needing to be referred from primary and secondary facilities, smaller conditions, if not recognised as sinister and then referred timeously, can result in advanced disease at index presentation. Many participants felt they were sent back and forth between healthcare facilities and not seen as urgent enough to be referred on. Frustration was apparent for those who felt they were given an elective date and for those who felt they were kept at base hospitals without any escalated treatment.

One participant exclaimed that they had gone to the doctors for a checkup:

‘… but they didn’t give me treatment; they just gave me a letter.’ (Participant 6)

Concern mounted as participants felt their condition worsened, but no perceived action from the community healthcare had occurred. One participant reported:

‘[*I was sent*] back and forth, and it’s growing, growing, growing.’ (Participant 2)

Another participant explained that he delayed because he simply waited for his elective date:

‘I was waiting for them. They give me appointments in September, so I was waiting for my appointment.’ (Participant 12)

A few participants felt that the healthcare system was too overwhelmed with too many unwell patients, and they felt one can get lost in the system. One participant reported, when asked why he took so long to get to the referral hospital:

‘They [*hospital*] are taking long. I don’t know what to say. Most of the people there are sick. So, you don’t know if you’re first or last.’ (Participant 24)

It appears that the initial presentation of the disease at the primary health facility was not recognised as a carcinoma that required excision. This was evidenced by participants reporting that the eye was treated repeatedly with wound dressings, ointments or eye drops:

‘The clinic took long and then I was thinking maybe it’s gonna be better because they [*were*] giving me that medication.’ (Participant 23)

### Alternative treatment sought

Most participants did not question which form of healthcare to seek and exclusively sought allopathic medicine. While alternative treatment options were explored, it was mostly out of desperation or as an adjunct while waiting for the hospital.

Treatment was attempted by some at home. One participant was told to use the juice of a lemon. Another tried to clean it with pharmacy-bought saline water. As one participant:

‘Somebody told me that you must use a drop of lemon.’ (Participant 12)

Traditional healers were sought in a few circumstances for various reasons. One participant was in Zimbabwe without funds to return to South Africa. He said he used the traditional healer out of desperation but was aware that his eye was already advanced in the disease process. One participant’s translator described that he was given something:

‘more like water … to clean … [*but it did not help as the eye*] was already rotten by that time.’ (Participant 4)

One participant, still awaiting exenteration, requested if she could attend a traditional healer as an alternative, as she was scared to lose her eye. Another participant expressed his scepticism regarding traditional healers:

‘Ah I did go there. They tell me long stories … they say it’s the ancestors, ancestors, and I don’t believe in them too much.’ (Participant 18)

His main reason for attending the traditional healers was to appease his mother, who felt this was a generational curse passed down through the forefathers:

‘She [*his mother*] said, let’s go and check. Maybe something passed down from ancestors … and we must go and check. Maybe the ancestors have a problem with you.’ (Participant 18)

The traditional healer gave alternative treatments:

‘… candles to light and pray upon them … to my father to ask him to forgive me.’ (Participant 18)

One participant told us he went to the traditional healer to get a better understanding of his condition while waiting for results from the hospital:

‘I can say I did go, because you know if you don’t understand what is happening. So, I did go after waiting for results.’ (Participant 24)

The son of one participant, who helped translate, explained that they went as part of their cultural obligations:

‘According to our kind, according to mine, we thought it’s about things of the ancestors.’ (Participant 21)

They sought the traditional healer advice primarily prior to medical advice. The advice received was that the eye condition was a result of the ancestors calling her to be a healer:

‘They said it’s a traditional thing. We need to go for initiations … it’s about the ancestors. We must go to the initiations to be a healer.’ (Participant 21)

### Stigma and fear

While many participants were unperturbed by their appearance, many felt that they would be viewed differently in society after having had this surgery. Some felt the stigma closer to home than others. Family members had not seen the participants without a bandage or eye patch over where the eye used to be, and they were fearful as to how the family would respond to them.

One participant told us with the help from the translator that he was scared how his family would perceive him after surgery:

‘… it won’t sit well with them … now they’re going to take out the eye, and the family, how are they going to look at him.’ (Participant 5)

One participant was saddened when she told the story of her grandchild’s fear at seeing her:

‘He’s crying, “Mama where is your eye?”’ (Participant 20)

Other participants felt the cruel withdrawal from their community, and many felt the need to isolate themselves. One participant’s son told of his mother:

‘It affected her because many people left us because they didn’t know what was going on … even always staying indoors … she was scared people will see what happened to the eye.’ (Participant 21)

Another participant also felt the need to isolate himself, as told through the translator:

‘He always separated himself from others … they were always staring at him, and it seems there’s something, they’re scared to be next to him.’ (Participant 22)

Additionally, patients expressed fear that they had to lose one eye:

‘It’s just stress. If you was having your two eyes … now you’re left with one.’ (Participant 12)

One participant was visibly upset when he spoke of the stigma of losing one eye, and how he was seen differently by his community after surgery:

‘You know how black people are; they can judge you for anything because they know me. I was having two eyes that time, but now … I’ve got this thing.’ (Participant 23)

### Relief and hope

While those who were still awaiting surgery expressed fear and hopelessness, as well as immense pain, the patients who had successful, curative surgery were relieved and could face the future with new hope. One participant’s son summarised it:

‘After the operation, it’s better because she can see there is a way forward … hope is there after the operation.’ (Participant 21)

Many were motivated by their family and felt they needed to be strong for them:

‘I have to be positive; I have to be strong for my son.’ (Participant 17)

Several participants now had a new lease on life and were adapting to their new normal. One participant summarised it:

‘I was relieved after they took the eye off, because I was me … for the first time there was no pain. The only thing that bothers me is I don’t have another eye … I used to have two eyes, which means it’s going to be a new me. I was not born with one eye, but I’m getting there slowly but surely. I’m relieved.’ (Participant 3)

## Discussion

Our primary objective was to establish which experiences and situations lead to delayed presentation. This led to the question, what was the lived experience of people living with periorbital carcinoma requiring OE? We explored the key themes through thematic analysis. The feeling that this was an insignificant condition and was ignored or not tended to timeously aligned with the work of Sharma et al.^9^, who found sometimes meeting the basic needs of life was more critical and that most patients only consulted a doctor when symptoms became too distressing or if they impacted day-to-day activities. Some individuals were just oblivious to the seriousness of the condition, thinking the mass would go away and, as Gichuhi et al.^11^ described, simply lacked the perceived need for treatment. While in the closed-ended questions, 76% of the patients said the mass was not urgent, the findings from the qualitative data were contradictory. Possible reasons for this may be a language barrier, with participants not fully understanding the question.

While financial constraints in a largely unemployed sample size are visible, it is also important to remember the financial implications for participants who must attend the clinic frequently and travel great distances to the hospital. Money was not only required for transport costs, but participants also required food for the long journey. Food security was often more of a priority, with participants spending their money on food, rather than on healthcare. Employment in the informal sector meant that patients stayed away for fear of loss of income during this period of health-seeking behaviour, as described by Gichuhi et al.^11^ Many were supported financially by family members, but those who were isolated with no family felt dread at having to borrow money from strangers who were often unwilling to give them money.

Delays caused by the clinic or hospital were often because of an overburdened healthcare system and participants having to wait for elective review dates. Our analysis identified that often the initial presentation at referral centres were not recognised as suspicious and so treated insufficiently with ointments and wound dressings. Some participants felt there were delays in waiting for results; others were unsure why there were delays. One participant felt that, had he had money to see a specialist, he would have been helped sooner.

In our context, participants were in favour of allopathic medicine and chose it above traditional medicine. However, culturally some felt the need to appease living relatives and the ancestors. Traditional healers were also sought as an adjunct while awaiting results, but still primarily relying on the treatment from allopathic medicine. This contrasted with Kishore et al.,^10^ who found that the population in Delhi, India, sought it as a last resort.

Hopelessness and fear associated with the diagnosis of cancer were evident. However, participants seemed more conflicted and distraught at the prospect of losing an eye. Some felt this way as the eye was still able to see. Others were worried about how they would be perceived by their family and community after the eye removal surgery. Isolation and alienation were a defensive method employed by the participants for fear of rejection, and often the community would exclude and judge them by not sitting near them or avoiding them.

A tumour invading ocular tissues and bone is a very painful condition. Physical relief from pain after the OE was a feeling reported by many participants. Emotional relief and acceptance of the condition and loss of an eye were also perceived by those who had complete, curative surgery. New hope and gratitude for life were expressed; participants felt they could see a future for themselves. Many planned to start working again, requesting referral for ocular prothesis and enquiring about grooming themselves while caring for a healing facial wound where the eye once was.

### Limitations

This study is not without limitations. Recordings were sometimes taken in a loud and busy clinic, which made for poor audio recordings at times. Interviews were often interrupted by nursing staff or other patients. The translators were different nursing staff each time and did not always explain well what the patient said. With the more sensitive questions, male patients were more reluctant to be vulnerable about their feelings compared to their female counterparts.

### Strengths

This appears to be the first study conducted in South Africa that looks to understand the lived experience of people who delayed presentation for an orbital mass requiring exenteration.

## Conclusion

Most participants acknowledged that they initially ignored the eye and its enlarging size, and that they only accepted that this was not going away when the eye’s condition worsened. Pain was a major motivator. However, once symptoms culminated in healthcare-seeking behaviour, participants felt they were then delayed awaiting clinic dates or biopsy results. Financial struggles meant participants lacked the freedom to engage in healthcare as they wished. Often there were more pressing things that took precedence, such as food and basic human survival. Loss of income was a double-edged sword, as many delayed treatments for fear of not getting paid, but ultimately many lost their jobs as they became no longer able to fulfil their duties with one eye. Many male participants experienced humiliation that, as former breadwinners, they were no longer able to provide for their families because of this condition. Traditional healers were pursued while waiting for results or to try to clarify esoteric questions. However, they were not the first line for accessing healthcare. Stigma surrounding the loss of an eye and the alienation received from the participants’ communities had a significant impact on their self-esteem. Many overcame these difficulties and chose to live their lives positively and establish a new normal for their lives. Hope and plans were what motivated those with complete curative surgical excision to carry on and to find the strength to support their families and themselves.

Recommendations to try to prevent delayed presentation for OE have both simple and layered complexities. Training should be offered to primary healthcare staff to identify the symptoms and signs of OSSN early and refer timeously to tertiary health facilities. Socio-economic and financial support to the patient is far more complex and not easily overcome. Once the patient is at the referral centre, one should endeavour to complete as many tests and investigations as possible. This is done to try to avoid multiple clinic visits. Counselling and emphasising the importance of follow up and the consequence and morbidity of the disease process should be explained.

## References

[1] Gichuhi S, Sagoo MS, Weiss HA, et al. Epidemiology of ocular surface squamous neoplasia in Africa. Trop Med Int Health. 2013;18(12):1424–1443. 10.1111/tmi.12203 24237784 PMC4440345

[2] Nagaiah G. Ocular surface squamous neoplasia in patients with HIV infection in sub-Saharan Africa. Curr Opin Oncol. 2010;22(5):437–442. 10.1097/CCO.0b013e32833cfcf9 20639761 PMC4209293

[3] Cicinelli MV, Marchese A, Bandello F, et al. Clinical management of ocular surface squamous neoplasia: A review of the current evidence. Ophthalmol Ther. 2018;7(2):247–262. 10.1007/s40123-018-0140-z 30030703 PMC6258579

[4] Shields CL, Chien JL, Surakiatchanukul T, Sioufi K, Lally SE, Shields JA. Conjunctival tumors: Review of clinical features, risks, biomarkers, and outcomes – The 2017 J. Donald M. Gass Lecture. Asia-Pacific J Ophthalmol. 2017;6(2):109–120. 10.22608/APO.201710 28399347

[5] Pe’er J. Ocular surface squamous neoplasia: Evidence for topical chemotherapy. Int Ophthalmol Clin. 2015;55(1):9–21. 10.1097/IIO.0000000000000050 25436490

[6] Martel A, Baillif S, Nahon-Esteve S, et al. Orbital exenteration: An updated review with perspectives. Surv Ophthalmol. 2021;66(5):856–876. 10.1016/j.survophthal.2021.01.008 33524457

[7] Gerring RC, Ott CT, Curry JM, Sargi ZB, Wester ST. Orbital exenteration for advanced periorbital non-melanoma skin cancer: Prognostic factors and survival. Nat Publ Gr. 2017;31(3):379–388. 10.1038/eye.2016.218 PMC5350354 27768120

[8] Raman-Abdulla P. An evaluation of orbital exenteration at St. John Eye Hospital. Master’s thesis. Johannesburg: University of the Witwatersrand.

[9] Sharma G, Gupta S, Gupta A, Kalyani VC, Rohilla KK, Thozhuthungal Sreejeev A, Yanthan YP, Kumar U, Das B, Sharma G, Sehrawat A, Gupta M. Identification of factors influencing delayed presentation of cancer patients. Int J Community Med Public Health [Internet]. 2020 Apr;7(5):1705–10. Available from: https://www.ijcmph.com/index.php/ijcmph/article/view/6354

[10] Kishore J, Ahmad I, Kaur R, Mohanta PK. Beliefs and perceptions about cancers among patients attending radiotherapy OPD in Delhi, India. Asian Pac J Cancer Prev. 2007;9(1):155–158. 18439096

[11] Gichuhi S, Kabiru J, M’Bongo Zindamoyen A, et al. Delay along the care-seeking journey of patients with ocular surface squamous neoplasia in Kenya. BMC Health Serv Res. 2017;17(1):1–11. 10.1186/s12913-017-2428-4 28705204 PMC5512725

[12] Polit DF, Beck CT. Nursing research: Generating and assessing evidence for nursing practice. Philadelphia: Lippincott Williams & Wilkins; 2008.

[13] Braun V, Clarke V, Boulton E, Davey L, McEvoy C. The online survey as a qualitative research tool. Int J Soc Res Methodol. 2021;24(6):641–654. 10.1080/13645579.2020.1805550

